# Genomewide Dam Methylation in *Escherichia coli* during Long-Term Stationary Phase

**DOI:** 10.1128/mSystems.00130-16

**Published:** 2016-12-13

**Authors:** Lacey L. Westphal, Peter Sauvey, Matthew M. Champion, Ian M. Ehrenreich, Steven E. Finkel

**Affiliations:** a Molecular and Computational Biology Section, Department of Biological Sciences, University of Southern California, Los Angeles, California, USA; b Department of Chemistry and Biochemistry, University of Notre Dame, Notre Dame, Indiana, USA; Northwestern University Feinberg School of Medicine

**Keywords:** Dam methyltransferase, long-term stationary phase, SMRT, epigenetics, long-term survival, methylation

## Abstract

DNA methylation in prokaryotes is widespread. The most common modification of the genome is the methylation of adenine at the N-6 position. In *Escherichia coli* K-12 and many gammaproteobacteria, this modification is catalyzed by DNA adenine methyltransferase (Dam) at the GATC consensus sequence and is known to modulate cellular processes including transcriptional regulation of gene expression, initiation of chromosomal replication, and DNA mismatch repair. While studies thus far have focused on the motifs associated with methylated adenine (meA), the frequency of meA across the genome, and temporal dynamics during early periods of incubation, here we conduct the first study on the temporal dynamics of adenine methylation in *E. coli* by Dam throughout all five phases of the bacterial life cycle in the laboratory. Using single-molecule real-time sequencing, we show that virtually all GATC sites are significantly methylated over time; nearly complete methylation of the chromosome was confirmed by mass spectroscopy analysis. However, we also detect 66 sites whose methylation patterns change significantly over time within a population, including three sites associated with sialic acid transport and catabolism, suggesting a potential role for Dam regulation of these genes; differential expression of this subset of genes was confirmed by quantitative real-time PCR. Further, we show significant growth defects of the *dam* mutant during long-term stationary phase (LTSP). Together these data suggest that the cell places a high premium on fully methylating the chromosome and that alterations in methylation patterns may have significant impact on patterns of transcription, maintenance of genetic fidelity, and cell survival.

**IMPORTANCE** While it has been shown that methylation remains relatively constant into early stationary phase of *E. coli*, this study goes further through death phase and long-term stationary phase, a unique time in the bacterial life cycle due to nutrient limitation and strong selection for mutants with increased fitness. The absence of methylation at GATC sites can influence the mutation frequency within a population due to aberrant mismatch repair. Therefore, it is important to investigate the methylation status of GATC sites in an environment where cells may not prioritize methylation of the chromosome. This study demonstrates that chromosome methylation remains a priority even under conditions of nutrient limitation, indicating that continuous methylation at GATC sites could be under positive selection.

## Abstract (precis)

While it has been shown that methylation remains relatively constant into early stationary phase of *E. coli*, this study goes further through death phase and long-term stationary phase, a unique time in the bacterial life cycle due to nutrient limitation and strong selection for mutants with increased fitness. The absence of methylation at GATC sites can influence the mutation frequency within a population due to aberrant mismatch repair. Therefore, it is important to investigate the methylation status of GATC sites in an environment where cells may not prioritize methylation of the chromosome. This study demonstrates that chromosome methylation remains a priority even under conditions of nutrient limitation, indicating that continuous methylation at GATC sites could be under positive selection.

## INTRODUCTION

Prokaryotes covalently modify their chromosomal DNA in several ways, the most common being methylation through base-specific methyltransferases (1). Methylation reactions occur at the N-6 position of adenine (methylated adenine [meA]) and the C-5 and N-4 positions of cytosine (m5C and m4C, respectively) using *S*-adenosylmethionine (SAM) as the methyl donor. These three modifications are common throughout the gammaproteobacteria (2).

In *Escherichia coli* K-12, the primary methyltransferases are DNA adenine methyltransferase (Dam), DNA cytosine methyltransferase (Dcm), and the M.EcoK restriction/modification methyltransferase. Dam targets 5′-GATC-3′ sites (of which *E. coli* K-12 strains have ~37,450 on both strands), methylating the N-6 position of adenine. Dam sites are slightly overrepresented in the *E. coli* genome, but they are specifically overrepresented in promoters (3). In fact, some transcription factor binding sites contain the GATC motif within their consensus sequences, including those binding the catabolite repressor protein (CRP) and the fumarate nitrate reductase (Fnr) regulator (3), potentially indicating an overlap in regulation. There are approximately 130 molecules of Dam in the cell during logarithmic phase growth and Dam moves along the chromosome processively, methylating ~55 GATC adenines before it releases from the DNA (4). Dcm is responsible for methylating the second cytosine (m5C) at ~24,000 sites with the sequence 5′-CC(A/T)GG-3′. Dcm sites are overrepresented in the genome, suggesting that they have been selected for over time (5). M.EcoK methylates the adenine at the N-6 position in the recognition sequences 5′-AAC(N6)GTGC-3′ and 5′-GCAC(N6)GTT-3′ (N6 is NNNNNN) of which there are ~1,000 sites on the chromosome. These recognition sites are not overrepresented in the genome, randomly occurring ~every 8 kbp (6). There is a fourth methyltransferase, YhdJ, encoded on the *E. coli* K-12 genome that is not expressed under standard laboratory conditions, but it has been shown to methylate 5′-ATGCAT-3′ sequences when overexpressed (7).

While the roles methylation plays in cells are broad (reviewed in references 1, 8, 9, 10, and 11), methylation by DNA adenine methyltransferase (Dam) (12) in *Escherichia coli* is necessary for proper cell cycle timing through methylation of *oriC* (13, 14), mismatch repair accuracy through discrimination between parental and newly synthesized DNA strands (15–17), regulation of transcription of a number of genes, including virulence factors (10), and regulation of transposition (18). Due to its role in regulation of transcription (4, 12), methylation is considered an epigenetic regulator (2, 9, 12).

This study focuses on methylation by Dam because of its known physiological roles within the cell. Methylation through Dam has been shown to vary depending on the environment (19). However, methylation of the genome has not been measured, as cells experience all five growth phases in rich media. Here we have assessed methylation levels throughout early (8 to 16 h of incubation) and late stationary phase (24 to 48 h of incubation), postdeath phase (72 h of incubation), and long-term stationary phase (LTSP) (96 to 120 h of incubation), which occurs after death phase (20). Methylation during exponential phase was not investigated in this study, as hemimethylation of the chromosome will be occurring in asynchronously dividing cells. LTSP is unique in that populations that survive death phase begin to actively replicate, although at a lower rate than occurs during exponential phase. This period of the bacterial life cycle encompasses many physiological and environmental changes that reflect what *E. coli* may experience in more natural environments (20–25). Using single-molecule real-time (SMRT) sequencing, we determined whether *E. coli* prioritizes methylating its genome while in LTSP when cells are under nutrient limitation but subpopulations of cells are beginning to replicate again. Subpopulations of cells during LTSP are known to be replicating because of the expression of the growth advantage in stationary phase (GASP) phenotype, where populations that have been aged in batch culture can outcompete parental strains (20, 25). Although cell density measurements remain the same in LTSP, the population is dynamic as selective sweeps, where initial populations are essentially replaced, are occurring under these conditions.

## RESULTS

### GATC methylation is nearly complete throughout 5 days of incubation.

**FIG 1:**
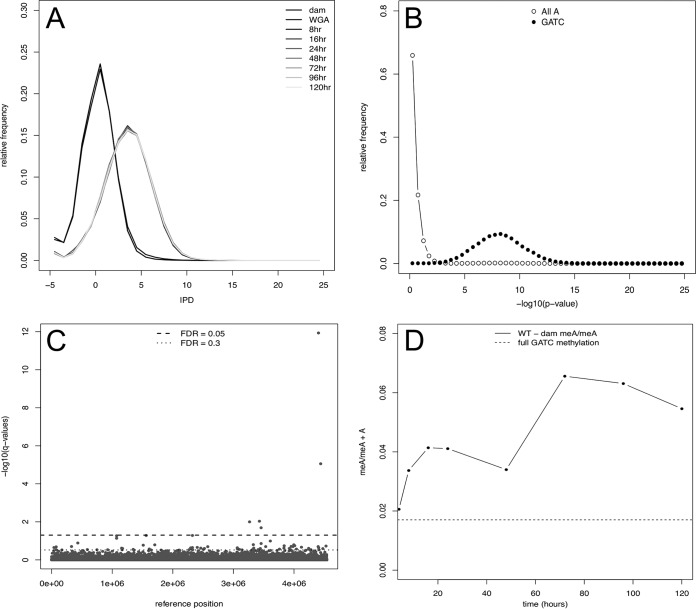
Virtually all GATC sites are methylated across time points**.** (A) The distribution of IPDs for each time point (8, 16, 24, 48, 72, 96, and 120 h) and negative-control samples (*dam* mutant strain and whole genome amplified [WGA]). The two distributions centered at 0 (black lines) are the negative controls (*dam* and WGA), while the light gray lines represent the time points. (B) A representative figure of the distribution of *P* values from a *t* test comparing the 8-h time point with the *dam* negative control. Open circles show the –log_10 _*P* values of adenines that are not located within a GATC site. Closed circles are the –log_10_ *P* values of adenines located within a GATC site. Panel B shows a representative time point, but every time point assessed is nearly identical (data not shown). (C) *q* values of a one-way ANOVA to determine significant temporal changes of IPD distributions. The dashed line represents an FDR of <0.05, while the dotted line and above represent an FDR of <0.3. (D) Liquid chromatography-mass spectroscopy (LC-MS) detection of methylated adenine over 5 days of incubation in LB. The black line represents the fraction of methylated adenine (meA) over total adenine over 5 days, performed in triplicate with biological duplicates. Since the LC-MS method cannot distinguish between methylated adenines located within GATC sites versus the total chromosome, levels of meA/total A may surpass expected levels due to nonspecific methylation by Dam, which has been reported previously to occur at 5′-GACC-3′, 5′-HATC-3′ (H = A, T, or C), and 5′-GATT-3′ sequences (56). Propagated error across samples (two biological samples with two replicates) was 6.8% (8 h), 13.8% (16 h), 14.7% (24 h), 5.9% (48 h), 43.9% (72 h), 5.8% (96 h), and 27.9% (120 h).

Previous work focusing on both logarithmic phase growth and early stationary phase (no more than 24 h) has shown that GATC sites in the gammaproteobacteria are methylated by Dam to near completion (26–28). To date, there has yet to be a characterization of methylation levels through late stationary phase, through death phase, and into long-term stationary phase. Although replication does not occur during early stationary phase, after death phase as cells transition into LTSP, growth of subpopulations resumes, giving rise to the possibility that methylation changes could occur at this time. As shown in Fig. 1A, Box-Cox-transformed interpulse duration (IPD) distributions for wild-type (WT) strains harvested over a 5-day time course are essentially the same, indicating equal levels of methylation at almost all GATC sites through all phases of growth. IPD values are a kinetic measurement of the incorporation of a nucleotide by DNA polymerase that is used to determine whether a modification such as methylation exists on the DNA template. Here, larger IPD values are associated with the presence of modifications on the genome as the incorporation of thymine across from a modified adenine causes a delay in the kinetics of incorporation. IPD values include all 37,452 GATC sites with a coverage of ≥20. Two control samples are also shown, reflecting chromosomal DNA isolated from a *dam* mutant strain that is incapable of GATC methylation, and a genomic sample randomly amplified by PCR which is devoid of any modification at all sites. IPD profiles of the two control samples differ significantly (false-discovery rate [FDR] of <0.05 by two-tailed *t* test) from all WT samples across all time points, reflecting an incomplete level of methylation.

All raw IPD values were Box-Cox transformed (29, 30) to a normal distribution, and time point samples were compared to those of the *dam* control in a two-sided *t* test to determine whether there were any sites where the IPD distribution differed significantly from that of the *dam* control. The data presented in Fig. 1B demonstrate that *P* values associated with the GATC motif are highly significant compared to the *dam* control, whereas *P* values associated with adenines not located within the GATC motif (All A) are not. Taken together, this analysis indicates that all sites possess detectable methylation at all times in WT samples. Further, the data indicate that almost all GATC sites are eventually fully methylated throughout long-term stationary phase. To confirm the SMRT sequencing results, a liquid chromatography-mass spectrometry (LC-MS) method using acid hydrolysis that can distinguish methylated from unmethylated adenine in total genomic DNA was developed. While this method can measure the ratio of methylated to nonmethylated adenine, it cannot determine GATC or site-specific methylation from hydrolyzed nucleic acids. Consistent with the SMRT sequencing results, LC-MS provides independent confirmation that in WT cells, virtually all Dam sites are methylated compared to a *dam* mutant strain, throughout 5 days of incubation (Fig. 1D). Comparing meA levels through LC-MS in a *dam* mutant and a WT strain allows for any background methylation through M.EcoK to be subtracted and ensures that the meA we detect is from Dam activity.

### GATC methylation levels change over time within the population at several sites.

**FIG 2:**
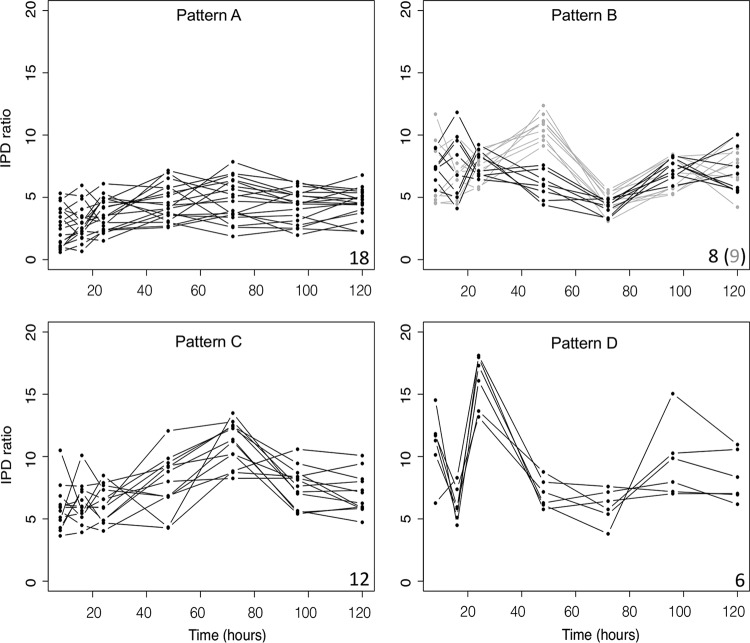
Four major patterns identified through hierarchical clustering plotted by IPD ratio over time**.** Pattern A contains sites where the IPD ratio is stable from 8 to 16 h and then gradually increases over time. Pattern B contains sites where the IPD ratio generally declines from 24 to 72 h (with a subpopulation that increases at 48 h) and then increases up to 120 h. Pattern C contains sites where IPD ratios reach the highest value at 72 h and then decrease. Pattern D sites have the highest IPD ratios at 24 h with a subsequent increase after 72 h. Numbers in the bottom right-hand corner in each panel indicate the number of sites with each pattern (grey number in parentheses represents the grey subpopulation in pattern B).

Although we determined that virtually all GATC sites show a significant difference in meA compared to the *dam* mutant strain throughout the time course, we wanted to determine whether any specific sites show quantitative changes in methylation across the population over time. We focused on sites where the IPD distributions change significantly (FDR < 0.3) over time and identified 66 GATC sites. Of these 66 sites, ~20% are located in noncoding regions, including within known promoter or regulatory regions (Fig. 1C). Many of the sites identified are located within genes that encode proteins involved in transport or transcriptional regulation or are located in the inner membrane (Table 1), and several of these sites were recently identified as being hypomethylated (28). Greater than 20% (14/66) of the sites are located within genes known to be regulated by CRP-cyclic AMP (cAMP) which binds a consensus sequence that contains GATC. Hierarchical clustering analysis of the 66 GATC sites based on IPD ratios (mean IPD sample/mean IPD *dam* at a specific site) identified four broad patterns of change (Fig. 2; see Fig. S1 in the supplemental material): pattern A, sites where the IPD ratio is low and constant between 8 and 16 h and then gradually increases over time; pattern B, sites where the IPD ratio generally declines from 24 to 72 h (with a subpopulation that increases at 48 h) and then increases up to 120 h; pattern C, sites where IPD ratios increase up to 72 h and then decrease through 120 h; and pattern D, sites where the greatest IPD ratio occurs at 24 h and 120 h.

**TABLE 1:** Gene Ontology term enrichment analysis of the 66 significant GATC sites^a^

GO term	No. of genes	Genes^b^
Transmembrane transport	16	*abgT*, *acrE*, *atpB*, *fadR*, *fecE*, *fhuC*, *glnP*, *hslU*, *kdgT melB*, *mrcA*, *mtlA*, *parE*, *tbpA*, *yhhS*, *yjhB*
Nucleoside phosphate binding or nucleotide binding	14	*ackA*, *bcsA*, *fecE*,* fhuC*,* hslU*, *hyfR*, *ldhA*, *nadR*, *nirB*, *parE*, *pheT*, *tbpA*, *yihV*, *yihU*
Regulated by CRP-cAMP	14	*ackA*, *acrE*, *bhsA*, *caiB*, *fecE*, *fhuC*, *hyfR*, *malP*, *melB*, *metH*, *mtlA*, *nirB*, *nuoE*, *yhhY*
Sulfur compound metabolic or biosynthetic process	11	*ackA*, *metH*, *nadR*, *parE*, *pheT*, *pta*, *thiE*, *yfaU*, *yigB*, *yihV*, *yihU*
ATP binding/adenyl ribonucleotide binding/ATPase activity	10	*ackA*, *fecE*, *fhuC*, *hslU*, *hyfR*, *nadR*, *parE*, *pheT*, *tbpA*, *yihV*
Magnesium ion binding	5	*abgT*, *atpB*, *glnP*, *kdgT*, *melB*
Regulated by Fur	4	*ackA*, *metH*, *pta*, *thiE*
Acetate metabolic process	4	*fecE*, *fhuC*, *metH*, *yhhY*
Regulated by NanR	3	*nanA*, *nanC*, *yjhB*
Regulated by CreB	3	*ackA*, *parE*, *pta*

a Enrichment analysis was performed by ecocyc.org (Materials and Methods), and *P* values are based on Fisher’s exact test and are considered significant if *P* < 0.1. GATC sites that lie within or upstream of genes that show significant changes in methylation over time are enriched for specific GO terms.

b Genes that appear in more than one category are underlined.

10.1128/mSystems.00130-16.1 Figure S1  Hierarchical clustering of IPD ratios by time point. Each number represents the genomic position of the significant site (q value of <0.3). Here we distinguished four major patterns (patterns A to D) of IPD ratios. The patterns are indicated by color as follows: pattern A (blue), pattern B (orange), pattern C (green), and pattern D (violet). Download Figure S1, TIF file, 0.1 MB. Copyright © 2016 Westphal et al. 2016 Westphal et al. This content is distributed under the terms of the Creative Commons Attribution 4.0 International license.

### Methylation of three GATC sites may be involved in regulating sialic acid catabolism.

**FIG 3:**
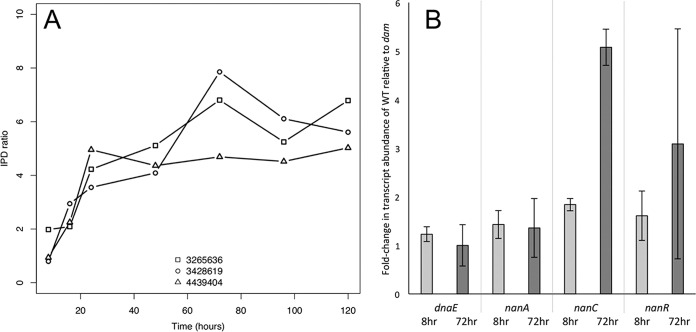
GATC sites where methylation changes over time correspond to a change in gene expression in a *dam* mutant. (A) IPD ratios of GATC sites with an FDR of <0.05 and involved in sialic acid catabolism across 5 days of incubation. The IPD ratio is the mean IPD of the sample at a specific position divided by the mean IPD of the control (*dam* mutant) at that same position. Therefore, an IPD ratio of 1 indicates that the means at that position are equal. The positions in the chromosome are shown in the legend (e.g., 3265636). (B) Transcript abundance fold change between WT and *dam* strains after 8 and 72 h of incubation in LB. Each bar represents an average of three biological replicates, and the error bars show the standard deviations. The *dnaE* strain is a negative control that is known not to be regulated through Dam methylation (and shows a fold change ratio of ~1). Note that transcription of *nanA*, whose promoter region does include GATC sites, does not change with the loss of Dam methylation, suggesting that other regulatory mechanisms are at play.

As a “proof of concept,” we chose to focus on three GATC sites with highly significant (FDR < 0.05) and similar patterns of methylation whose role in transcriptional regulation of these particular sites is well characterized (31–37). These sites were associated with genes involved in *N*-acetylneuraminic acid (sialic acid) uptake and catabolism (38, 39). Sialic acid is a monosaccharide derived primarily from eukaryotes. While *E. coli* does not produce sialic acid, it is able to metabolize this compound (36, 38, 40). Figure 3A shows the IPD ratios (mean IPD sample/mean IPD *dam*) of each of these sites at each time point. While an individual GATC site is either methylated or unmethylated, in a population of cells that site may be methylated in different proportions. Therefore, the significant differences between time points shown here reflect a change in the proportion of methylation over time within the population. Each of these three sites has been reported as being protected from methylation and demonstrated to play a role in regulating sialic acid catabolism and/or transport (31). To demonstrate a link between our methylation data and potential Dam regulation, we determined the expression levels of the genes downstream of the GATC sites at the time points where the greatest difference in IPD ratios was observed. Figure 3B shows the fold change in expression levels between a WT and *dam* mutant strain at both 8 and 72 h of incubation. Although *dam* mutants may have pleiotropic effects, no significant fold changes in gene expression were observed between the WT and *dam* strains in any gene assayed after 8 h of incubation, but by 72 h, gene expression of the *nanC* and *nanR* genes is 3- to 5-fold lower in the *dam* mutant, consistent with binding of the NanR repressor, whose consensus binding sequence occurs adjacent to GATC sites. We hypothesize that methylation at these sites may sterically block NanR from binding these sites. While work by others has not specifically shown that methylation at these sites blocks the binding of NanR, it has been shown here and by others that the presence of Dam increases transcription of *nanA* and *nanC* (3).

### Mutations in *dam* are selected against within heterogeneous populations.

**FIG 4:**
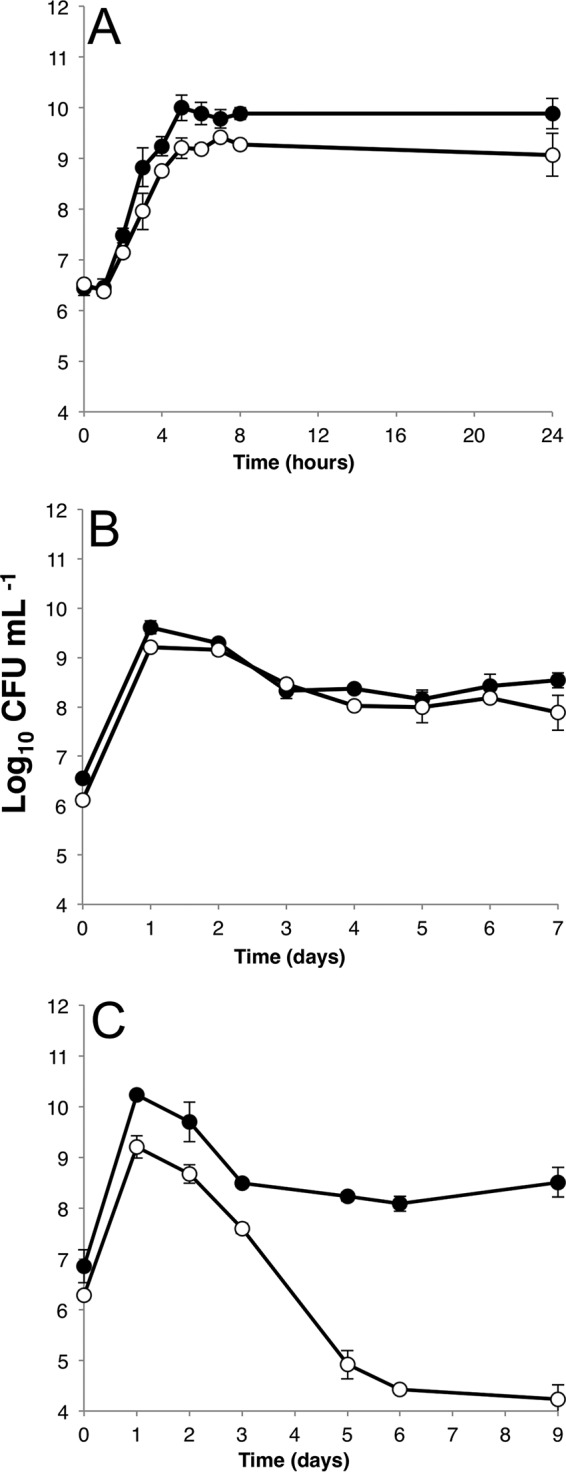
Growth of WT and *dam* strains in monoculture and competition. (A) Twenty-four hours of growth in LB monoculture of the wild-type strain (black circles) and the *dam* mutant strain (white circles). (B) Seven days of incubation in LB monoculture of the wild-type strain (black circles) and the *dam* mutant strain (white circles). (C) Coculture of WT strain (black circles) and *dam* strain (white circles) in LB. Strains were inoculated at equal densities and competed for 9 days. All experiments are biological replicates done in triplicate; error bars represent the standard deviations.

Given that expression of certain genes appears to be sensitive to the overall methylation status of the promoter and the methylation of *oriC* has been shown to be essential for proper timing of DNA replication, we wanted to know whether *dam* mutants would have a competitive disadvantage within a heterogeneous population. In monocultures of *dam* mutant strains and WT strains, lag phase is nearly identical. However, during exponential growth, *dam* strains have a doubling time of 23.5 min, while WT strains double every 18 min. While WT strains reach stationary phase after 8 h of exponential growth, *dam* strains enter into stationary phase earlier, at about 6 h, and at a cell density ~10 times lower (Fig. 4A). Once cells enter stationary phase, the cell densities between WT and *dam* strains look remarkably similar (Fig. 4B). Both strains enter death phase at the same time and stabilize at ~10^7^ CFU/ml during long-term stationary phase. While strains that harbor lysogenic prophage may experience a loss of viability in the absence of *dam* (41), the strain of *E. coli* used here has no known lysogenic prophage. However, in direct competition, the fraction of *dam* mutants in the population decreases when competing with WT strains (Fig. 4C), indicating the importance of methylation to the survival of cells in a nonhomogenous environment.

## DISCUSSION

Methylation of the chromosome is important to many fundamental cellular processes. Because of this, it has been assumed that methylation is constant throughout growth, the hemimethylated state of the genome directly after replication and the origin of replication during exponential phase growth being important exceptions. Here, we show that most GATC sites are methylated in WT strains, from early stationary phase, and beyond death phase into long-term stationary phase. Although several significant differences are observed, it appears that the cell maintains complete methylation at GATC sites at virtually all times, even when subpopulations of cells in LTSP are replicating under starvation conditions. This finding might initially appear surprising given that methionine, cysteine, and aspartate are required for the synthesis of *S*-adenosylmethionine, the methyl donor for Dam, and cells experiencing LTSP are known to be starving for amino acids (42). However, once the population becomes fully methylated after log phase, SAM pools appear to be sufficient to allow full methylation of chromosomes resulting from replication after death phase. Additionally, while it is known that subpopulations are beginning to replicate after death phase, the depth of sequencing coverage in this study is insufficient to detect these subpopulations. For the small number of sites where the proportion of the cells with methylated GATC changes over time, we observe four general patterns of methylation.

GATC sites that experience a change in methylation over time are likely to be sites where proteins bind after a replication event or where DNA structure is not conducive to Dam binding, as there are no known demethylation mechanisms in *E. coli*. Whether a GATC site located in the promoter is methylated or not can determine whether the corresponding gene is expressed. Methylation of promoter regions has been shown both to induce expression by blocking repressor binding and to repress expression by hindering RNA polymerase from binding (8). Therefore, hypomethylated sites may cause changes in transcription. There is also the potential for local mutation frequency to change because MutH may cut the wrong strand during mismatch repair if there is no methylation to detect at the GATC site nearest to the mismatch. In this case, a hypomethylated GATC site would need to occur in a hypomethylated region greater than 1 kb in length to result in a lack of strand discrimination by MutH (43). There are ~400 sites on the *E. coli* chromosome where there is a gap of >1 kb between GATC sites, making this event unlikely, but possible.

The *nan* genes are responsible for catabolism of sialic acid into *N*-acetylglucosamine-6-phosphate (GlcNAc-6-P), which then enters into central metabolism after further processing by NagA and NagB (31, 39). Since *E. coli* K-12 cannot synthesize sialic acid, any source must be exogenous to the cell and of eukaryotic origin. When sialic acid is present in culture media, it is taken up by cells and binds NanR, causing it to dissociate from the chromosome, derepressing transcription of the *nan* operon (35). NanR binds within 5 bp of each of the three GATC sites we identified here; it has been speculated that full induction of each operon commences only when these GATC sites are fully methylated (34). Therefore, an increase in methylation at these sites over time may correlate with an increase in transcription of *nan* genes, assuming that methylation blocks the binding of NanR, which has not been confirmed experimentally. Future studies looking at total percentage of transcript abundance of *nanA* and *nanR* with RNA sequencing may help to determine whether methylation at these sites increases the transcription of these genes. Using a spectrophotometric sialic acid quantification kit (see Materials and Methods), we determined that LB contains ~120 nM sialic acid, a concentration reported to stimulate the induction of the *nan* operons due to the dissociation of NanR (33). Once NanR dissociates from the promoter, we hypothesize that Dam methylation may reduce its ability to rebind, as sialic acid concentrations decrease in the cell, leading to complete catabolism of this nutrient. Because nutrients can be limiting in the gut and sialic acid is present in the host system, it may be advantageous for cells to begin catabolizing sialic acid as soon as it is available. In fact, compared to WT strains, mutants lacking two other *nan* genes (*nanA* and *nanT*) have a competitive disadvantage when passed through the mouse gut (44). Therefore, in addition to serving as a source of nutrients, sensing the presence of sialic acid may be used as a signal to cells that they are located within a host. The biological relevance of this data indicates that further dissection of the time course methylation patterns may lead to the identification of additional Dam-regulated sites.

Nearly complete GATC methylation of the genome at all times may be important for cellular function, since methylation is maintained even under conditions of significant nutrient limitation in the culture environment. Even after death phase (after 48 h of incubation) when cells are surviving on detrital nutrients and growth of subpopulations of cells reinitiates, there is very little change in the methylation status of the genome. Monocultures of *dam* mutant strains are viable but do not reach the same density as WT strains after exponential growth (Fig. 4A and B). Additionally, *dam* strains decrease in density when directly competing with WT strains (Fig. 4C), indicating the importance of methylation to the survival of cells in a nonhomogenous environment and a strong selective pressure against *dam* mutants. This constant maintenance of methylation implies that the many phenotypes associated with a lack of methylation at GATC sites are too deleterious to be tolerated, particularly under conditions of stress. Whether there is a direct mechanism by which cells measure the amount of methylation present on the genome or there is negative selection at work against those cells that cannot methylate the entirety of their genome remains to be determined, but in order to be a member of the majority population in these cultures, cells must be able to essentially fully methylate their chromosomes.

## MATERIALS AND METHODS

### Strains and growth conditions.

All experiments were performed with strains derived from the *E. coli* K-12 W3110 lineage strain ZK126 (45). The *dam* mutant strain SF2601 was constructed by P1 transduction from strain GM3819, which carries a replacement of *dam* with a kanamycin resistance gene cassette (46). The *dam*-*16*::kan P1 lysate was transduced into strain ZK126 carrying pTP166 (*dam*^*+*^ plasmid; temperature sensitive; *dam* is necessary for P1 packaging) and then cured of pTP166. Replacement of the WT allele of *dam* with *dam-16*::kan was confirmed by PCR. Under coculture conditions, fresh overnight cultures of WT ZK1142, marked with nalidixic acid resistance (47) and *dam* strains were inoculated 1:1,000 (vol/vol) into the same test tube and monitored over time through viable cell counts in triplicate. Bacteria were cultured in 5.0 ml of LB broth, Lennox (Difco-BD) in borosilicate test tubes (18 by 150 mm) at 37°C with aeration in a TC-7 test tube roller (New Brunswick Scientific). Viable cell counts were determined by serial dilution and plating onto appropriate LB agar plates supplemented with 50 μg/ml kanamycin or 20 μg/ml nalidixic acid (48).

### SMRT sequencing.

Genomic DNA was extracted after 8, 16, 24, 48, 72, 96, or 120 h of batch culture incubation in test tubes using the DNeasy kit (Qiagen). The *dam* DNA control sample was extracted from *dam* mutant cells after 24 h of incubation. Whole genome amplified (WGA) controls were made from WT DNA extracted after 24 h of incubation and amplified using multiple displacement amplification (REPLI-g minikit; Qiagen). DNA was quantified using a NanoDrop 1000c spectrophotometer and the Qubit dsDNA (double-stranded) BR assay with a Qubit 2.0 fluorometer (Life Technologies, Inc./Invitrogen). Genomic DNA quality was assessed by running samples on a 0.7% agarose gel (data not shown). SMRT sequencing (Pacific Biosciences) was performed at the University of California, San Diego (UCSD) Institute for Genomic Medicine (IGM) Genomics Center with P4-C2 chemistry on a PacBio RS II DNA sequencing system using standard protocols.

### SMRT data analysis.

IPD distributions are shown in Fig. 1A. For *de novo* assembly of the reference genome, reads were included if they were greater or equal to a read score of 0.75 and a minimum length of 50 bp. Filtered subreads from each time point were combined to a total coverage of 600× for assembly with the hierarchical genome assembly process (HGAP2) with default parameters (49), resulting in a single contig. Filtered subreads for each time point had an average coverage of 67× with minimum coverage of 49× for the 48-h time point. The average read length across all time points was ~2.4 kb. Values of raw IPDs greater than 50,000 were considered outliers and discarded from all data sets. IPD statistical testing was done with Box-Cox-transformed IPD values and parametric testing. Python and R scripts for all SMRT analysis can be found at https://github.com/lwestphal/SMRT.

### Statistical analysis of SMRT sequencing data.

To perform statistical analysis, raw IPD values were Box-Cox transformed (29, 30) using the following equation:

$$\text{IPD}t=\frac{{\left(\text{IPD}+\alpha \right)}^{\lambda }-1}{\lambda }$$

Alpha and lambda parameters (α = 0.311, λ = 0.151) were chosen to optimize skewness and kurtosis distributions across all time points. The transformed IPD values were then centered at zero to allow for direct comparison between time points. Centering was accomplished by subtracting the mean IPD across reference positions from each IPD value, by reference position. In order to determine whether there was a significant difference in methylation across the time points compared to the negative control (*dam* mutant strain), a two-sided *t* test was performed across reference positions. This method differs from the PacBio RS_Modification_and_Motif_Analysis by using a biological negative control versus an *in silico* negative control. GATC sites that were not significant (sites where the distribution of IPDs in the WT were indistinguishable from *dam* mutant) were further tested in two ways: (i) by normalizing for equal coverage between *dam* and WT strains and (ii) by normalizing for equal coverage by random sampling and bootstrapping. To address changes in methylation across time, Box-Cox-transformed IPD values from all time points were tested with a one-way analysis of variance (ANOVA). *q* values were determined based on *P* values from the ANOVA using John Storey’s method in R (qvalue package) (50), and significance was based on an FDR cutoff of <0.05.

### Nonparametric statistics.

Statistical analysis was performed with both raw IPD values and Box-Cox-transformed IPD values. Due to the nonparametric distribution of raw IPD values, nonparametric statistical methods were used in addition to parametric testing. Methylation status, by GATC site, was determined with a Wilcoxon rank sum test using IPD distributions from *dam* strains as a negative control. Wilcoxon rank sum test was performed for each time point, at each reference position, and between whole genome amplified (WGA) data and *dam* data. Changes in methylation across time points were determined by a Kruskal-Wallis test. *q* values (false-discovery rate) were determined based on *P* values using John Storey’s method in R (q value package) (50), and significance was based on an FDR cutoff of <0.05. The results from the parametric and nonparametric statistical tests were similar with parametric one-way ANOVA identifying five significant sites versus six identified by Kruskal-Wallis test.

### DNA hydrolysis for mass spectroscopy analysis.

DNA was hydrolyzed to nucleobases using a protocol modified from references 51 and 52. Triplicates of 10 µg of dried genomic DNA was dissolved in 100 µl of 98% LC-MS grade formic acid (FA) (Fisher Scientific, Waltham, MA) sealed in glass tubes and hydrolyzed in a covered heat block at 90°C for 6 h. Hydrolysis was monitored by LC-MS as described below, and loss of genomic DNA was observed visually on a 0.7% agarose gel. After cooling, nucleobase hydrolysates were collected at the tube bottom and dried in a speed vacuum concentrator (Genevac, Stone Ridge, NY). Samples were stored at − 20°C until analysis (<2 days).

### LC-MS analysis.

Dried nucleobases were resuspended in 150 µl of 1% methanol (MeOH)− 0.1% FA and vortexed vigorously. Twenty microliters (~1.2 µg) was injected, in triplicate per genomic replicate (9 total per sample), onto an rapid separation LC (RSLC) ultrahigh-performance LC (UHPLC) system (Dionex, Waltham, MA) running at 400 µl/min. Samples were separated over an RSLC Polar Advantage II 2.2-µm column (2.1 mm × 100 mm) (Dionex) with a 10-min linear gradient from 0 to 60%  solvent B (solvent A is H_2_O plus 0.1% FA, and solvent B is MeOH plus 0.1% FA) (0.3 min at 0% solvent B, to 5 min at 60% solvent B, to 5.5 min at 80% solvent B, to 6.5 min at 80% solvent B, to 0% solvent B at 7 min to 0% solvent B at 10 min). All solvents were LC-MS grade (Fisher Scientific). MS acquisition was performed on a MicrOTOF-Q II electron spray ionization (ESI) time of flight (ToF) mass spectrometer (Bruker, Billerica, MA) as in reference 53. MS conditions were as follows: -500 V plate bias, +2,000 V capillary 5-Bar nebulizer 10-bar dry gasses (N_2_), capillary temperature of 180°C. MS spectra were acquired with a calibrated “Tune-low” method collecting data from 50 to 400 *m*/*z* summing two scans at 1 Hz. Samples were then diluted 5-fold and reinjected in triplicate to recover linearity for adenine. MS calibration was performed with nucleobase standards A, T, G, and C obtained from Sigma (St. Louis, MO) and N-6-methyladenine (meA) from Santa Cruz Biotechnology (Dallas, TX). Standard curves to determine dynamic range and limit of detection were performed using neat and standard addition into a matrix of *dam* genomic DNA which contains no meA. Retention time for A shifted slightly (0.2 min) compared to the neat curve. This is due to the fact that A elutes very close to the void volume and was most affected by sample composition. All five nucleobases (A, T, G, C, and meA) had linearity (*r*^2^ > 0.99) over 3 orders of magnitude, which is well within the requirements for this ratiometric analysis. Extracted ion chromatograms were performed ±50 millimass units (mmu) for A, T, G, C, meA on all samples at 136.0618 *m*/*z*, 127.0502 *m*/*z*, 152.0567 *m*/*z*, 112.0505 *m*/*z*, and 150.0774 *m*/*z* [M+H]^+^, respectively. Peaks were smoothed with a three-point Gaussian smoothing function and integrated using a Gaussian fit (Bruker, Billerica, MA). Peak areas and dilution corrected peak areas were exported to Excel, and methylation analysis was performed by calculating [meA]/([meA] + [A]) ratios for all samples per unit of DNA. *dam* genomic DNA spiked with various amounts of meA was used as a control. Ratios to Chargaff’s rules were also used to normalize signal responses for the nucleobases. Error is taken as the percent coefficient of variation (CV) (standard deviation/mean).

### Quantification of sialic acid in LB.

Sialic acid quantification was carried out in standard LB broth (Difco-BD) at full concentration (1×) and half-concentration (1/2×) with the sialic acid quantification kit (Sigma-Aldrich). This kit determines total *N*-acetylneuraminic acid (NANA) in the range of 1 to 200 nM. Quantification was performed in triplicate and reported as an average.

### Quantitative RT-PCR.

Gene expression was determined using quantitative reverse transcription-PCR (qRT-PCR). Total RNA was extracted from cultures after 8 or 72 h of incubation in batch culture using RNeasy and Bacteria RNAprotect (Qiagen). Samples were treated with DNase (Qiagen) on the column, and the absence of DNA was confirmed by PCR. Equal mass of total RNA was immediately converted to cDNA using the Tetro cDNA synthesis kit (Bioline) and then diluted to 25 ng/µl in nuclease-free water. quantitative PCR (qPCR) was performed using the No-ROX RT-PCR master mix (Bioline) with SYBR green on an Opticon 2 real-time PCR machine (Bio-Rad) using the Bioline standard protocol. The percentage of rRNA within total RNA samples remains constant even through LTSP (unpublished observation). Because there are no reference genes that maintain equal levels of expression throughout 5 days of incubation, relative expression was determined by the equation 2^Δ*C*(*T*)^, all threshold cycle (*C*_*T*_) values were normalized to the mass of total RNA added to each reaction mixture and primer efficiency. Primer sequences and concentrations are shown in Table 2. Expression values of *dam* cells were determined relative to WT expression at the two time points. Fold changes between time points and raw *C*_*T*_values are shown in Fig. S2 and Table S1 in the supplemental material.

**TABLE 2:** Primers used for qRT-PCR

Primer	Sequence	Concn (nM)
*dnaE* Forward	5′-TACCATTTCCACGTCAACGA-3′	600
*dnaE* Reverse	5′-CCCGGACATGATCAGTTTTT-3′	600
*nanA* Forward	5′-TGTTACATTGCCTGGCGTAG-3′	200
*nanA* Reverse	5′-TCTTTCAGCGCCTTAACGAT-3′	200
*nanC* Forward	5′-TCGTTACGACTGGAAAGCTTA-3′	200
*nanC* Reverse	5′-GCCCATTTCTTATGGTTTGC-3′	200
*nanR* Forward	5′-TTCTGCGGACACTATCATCG-3′	600
*nanR* Reverse	5′-CGTTGTTATCCAGCGACTGA-3′	600

10.1128/mSystems.00130-16.2 Figure S2  Fold change in expression between 8- and 72-h time points. The label above each bar specifies which gene is represented and whether that sample is from WT mRNA or *dam* mutant mRNA. Note that *dnaE* expression decreases ~50-fold between 8 h and 72 h of incubation but both the WT and *dam* samples show the same decrease in expression, as expected. Error bars represent the standard deviations of three biological replicates. Download Figure S2, TIF file, 0.7 MB. Copyright © 2016 Westphal et al. 2016 Westphal et al. This content is distributed under the terms of the Creative Commons Attribution 4.0 International license.

10.1128/mSystems.00130-16.3 Table S1  Critical threshold values from qRT-PCR. Download Table S1, XLSX file, 0.05 MB. Copyright © 2016 Westphal et al. 2016 Westphal et al. This content is distributed under the terms of the Creative Commons Attribution 4.0 International license.

### Determination of IPD ratio pattern formation.

GATC sites with a significant one-way ANOVA *P* value (*q* value < 0.3) were plotted as IPD ratios over time. Information regarding the position and context of these sites can be found in Table S2 in the supplemental material. To cluster patterns quantitatively, Euclidean distance measurements were taken between the IPD ratios of each time point and then hierarchically clustered by the Ward method (54). A dendrogram of the clustering of the sites is shown in Fig. S1.

10.1128/mSystems.00130-16.4 Table S2  Locations of the 66 GATC sites where methylation changes over time. Download Table S2, XLSX file, 0.03 MB. Copyright © 2016 Westphal et al. 2016 Westphal et al. This content is distributed under the terms of the Creative Commons Attribution 4.0 International license.

### Gene Ontology enrichment analysis.

To determine whether any molecular functions or global regulators were overrepresented in the 66 significant sites, Gene Ontology (GO) enrichment analysis was performed through the Smart Table functions at Ecocyc.org (55). Significance for enrichment was determined by Fisher’s exact test and considered significant if *P* values were <0.1.

### Accession number(s).

SMRT sequencing raw data files can be found at figshare (https://dx.doi.org/10.6084/m9.figshare.4168044.v1). Raw *C*_*T*_ values from gene expression data can be found in Table S1 in the supplemental material. Reference genome (ZK126) FASTA can be found at NCBI under accession no. CP017979. Raw and processed mass spectrometry data can be found at the MassIVE repository (accession no. MSV000080312). Scripts for data analysis can be found at https://github.com/lwestphal/SMRT.
