# Supplementary figures and images for: Genomewide Dam Methylation in Escherichia coli during Long-Term Stationary Phase
Source: mSystems. 2016 Dec 13;1(6):e00130-16. doi: 10.1128/mSystems.00130-16 (PMC5155068; doi:10.1128/mSystems.00130-16)

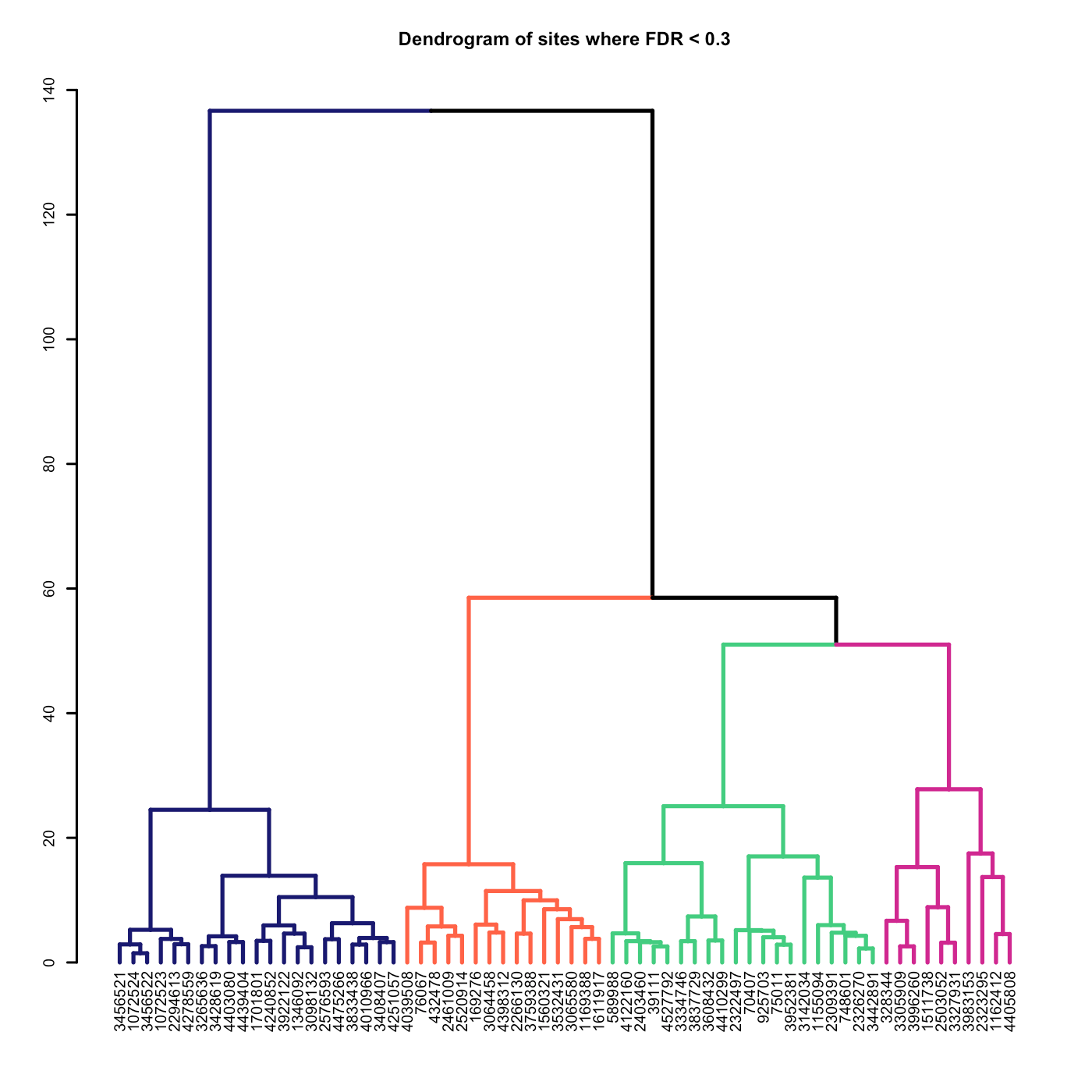

Supplement: Figure S1 [file sys006162070sf1.tif]

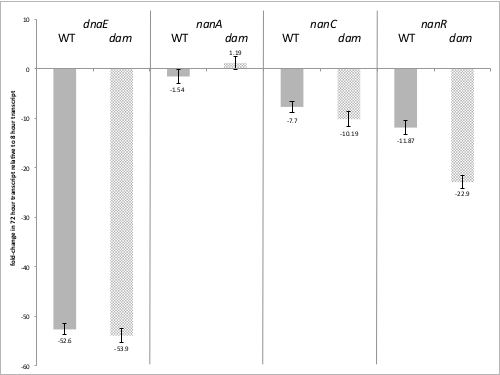

Supplement: Figure S2 [file sys006162070sf2.tif]
